# A Model for Secondary Monitor Unit Calculations of PBS Proton Therapy Treatment Plans

**DOI:** 10.14338/IJPT-18-00031.1

**Published:** 2019-03-21

**Authors:** Greg Schimke, Joseph Syh, Hsinshun Terry Wu

**Affiliations:** 1 Radiation Oncology, Willis-Knighton Cancer Center, 2600 Kings Hwy, Shreveport LA, USA

**Keywords:** proton therapy, pencil beam scanning, monitor unit calculation

## Abstract

**Purpose:**

This article summarizes a volume-based method by which secondary monitor unit (MU) calculations may be performed for pencil beam scanning, single field uniform dose (SFUD) proton therapy treatment plans.

**Materials and Methods:**

Treatment planning system (TPS) simulations were performed by using the local beam model to define relationships between planning target volume (PTV) characteristics and the MUs required to deliver a uniform dose for a given beam orientation. Relevant target attributes included volume, depth (ie, beam range), range-shifter air gap, and the projected area of the target volume in the beam's eye view (BEV). The proposed model approximates the PTV as a simplified cuboid region of interest as defined by its volume and BEV projected area. Output factors (cGy/MU) were then tabulated for the idealized geometry through TPS simulations using region of interests with a range of dimensions expected to be seen clinically. Correction factors were applied that account for differences between the PTV and the idealized conditions, and MUs for each beam were then scaled according to the measured spread out Bragg peak (SOBP) dose in water.

**Results:**

Our model was applied to various treatment sites, including pelvis, brain, lung, and head and neck. Monitor units prescribed by the TPS were compared to those predicted by using the model for 78 treatment beams. The total mean percentage difference for all beams was −0.2% ± 3.8%.

**Conclusion:**

This work demonstrates the potential for reasonably accurate secondary verification of MUs in pencil beam scanning proton therapy for SFUD treatment plans with the proposed method. Required inputs are few, and are readily accessible, facilitating automation and clinical application. Further investigation will expand the current model to accommodate a broader range of potential optimization problems, and intensity-modulated proton therapy treatment plans.

## Introduction

Pencil beam scanning (PBS) proton therapy does not lend itself to traditional monitor unit (MU) calculation methods used for passive scattering systems. Currently, to our knowledge, no commercial software exists for secondary calculation of MUs in PBS proton therapy and, with some exceptions, these calculations are not performed at most institutions. It is, however, a well-established practice in radiation therapy to understand and verify prescribed MUs by using an alternative method that involves the calculation of dose to a single reference point [1–3].

The value of 1 MU reflects a quantity of charge collected by the monitor chamber for a given irradiation, and may or may not be tuned to match to a clinically relevant quantity [4]. For passive scattering proton therapy systems, calculations of MUs are based on factors derived from the dosimetric characteristics of various spread out Bragg peak (SOBP) beams in conjunction with the compensator and snout required to deliver the prescribed dose to a single point [5]. In PBS proton therapy, however, treatments are delivered with thousands of individual magnetically steered pencil beams that are delivered in a polyenergetic spot pattern such that the target volume receives a uniform dose, and are almost exclusively created by using inverse optimization techniques. Pencil beam scanning systems are therefore not capable of delivering uniform volumetric reference fields without the use of inverse optimization, which complicates cGy/MU output characterization for the composite beam, even when the desired dose distribution is uniform. For this reason, it is common to first create a beam model, and subsequently validate absolute and relative dose distributions with deliverable treatment plans from the treatment planning system (TPS) [4, 6]. At our institution, validation of the beam model was performed in part by measuring the dose to a range of cuboidal volumes located at various locations in the phantom and irradiated to a uniform dose as prescribed by the TPS [6].

Similarly, the proposed model is based on cGy/MU output factors derived from simple, TPS-defined cuboidal treatment fields. Treatment plans were created and optimized with a standard single field uniform dose (SFUD) optimization goal to obtain output factors, and correction factors were applied on the basis of empirical relationships between the physical geometry of the target/beam combination and the beam output that is required to uniformly irradiate the target. Correction factors defined here are with respect to the beam range, and range shifter air gap. Calculated MUs were then scaled according to the measured dose in a water phantom at the center of the SOBP to arrive at the final result.

A comparable approach, described by Zhu et al [7], used a linear fit method to establish a relationship between the target volume and planned MUs exclusively for prostate targets. Our method, while inherently dependent upon the volume, also takes into account the shape and depth of the target, allowing it to be applied more broadly to other treatment sites.

Additionally, this approach differs from typical second-check calculations in that it does not require detailed beam delivery and spot information from the TPS, but rather determines MUs on the basis solely of the defined structures and beam orientation. It therefore can serve to verify or to predict prescribed MUs for SFUD treatment beams.

## Materials and Methods

### Formalism

Raystation (RaySearch Medical Laboratories AB, Stockholm, Sweden) TPS was used to identify and define empirical factor-based relationships between key geometric attributes of the target volume and MU output for the local beam model. The built-in python scripting interface was used to automate data retrieval and perform calculations. The resulting notation is analogous to that which is commonly used for conventional external beam MU calculations. Prescribed dose is taken to be point dose at the center of the SOBP region as measured in a water equivalent phantom from patient-specific quality assurance procedures. Off-axis ratios and inverse square correction factors were deemed negligible owing to the large effective source-axis distance of the PBS system, which is consistent with local commissioning beam data.

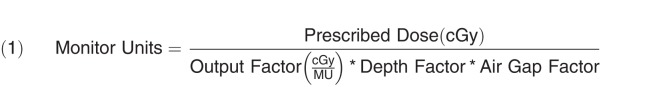



### Output Factor

**Figure 1.**
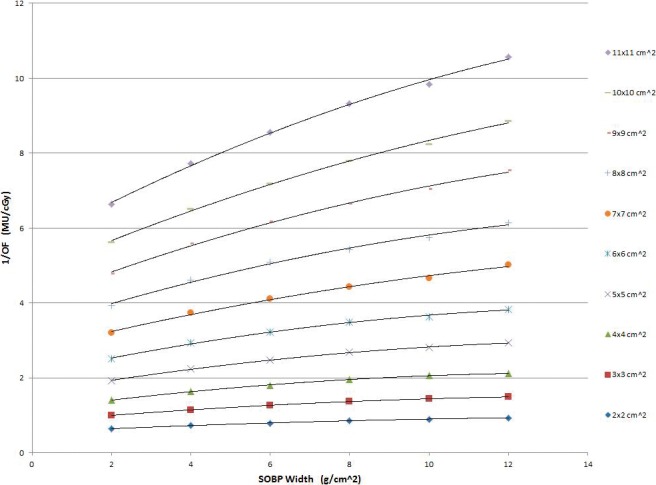
Reference output factors as a function of BEV projection area and SOBP width. Abbreviations: BEV, beam's eye view; MU, monitor unit; OF, output factor; SOBP, spread out Bragg peak.

**Figure 2.**
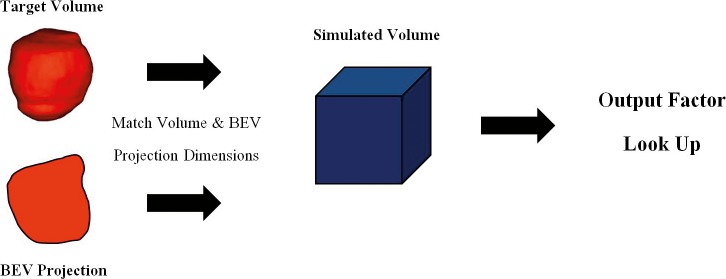
Flow chart illustrating the process of output factor computation. Abbreviation: BEV, beam's eye view.

Reference output factors (cGy/MU) shown in **Figure 1** were tabulated by using TPS simulations of cuboidal volumes and interpolated for the range of target dimensions expected to be seen clinically (up to 1500 cm^3^). A standard SFUD optimization goal was applied with the volumes located at a reference depth in water of 20 cm for open fields (this is effectively 15.9 cm for range-shifted fields owing to the presence of the 4.1-cm water equivalent thickness range shifter), and a reference air gap of 1 cm for range-shifted fields. Optimization settings for spot weighting and filtering, and the total number of iterations, were chosen to reflect typical practice at our institution. The collected data were then used to assign an output factor for each treatment beam from the volume and beam's eye view projection area of the target volume. A flow chart picturing the concept of output factor determination is shown in **Figure 2**.

### Depth Factor

**Figure 3.**
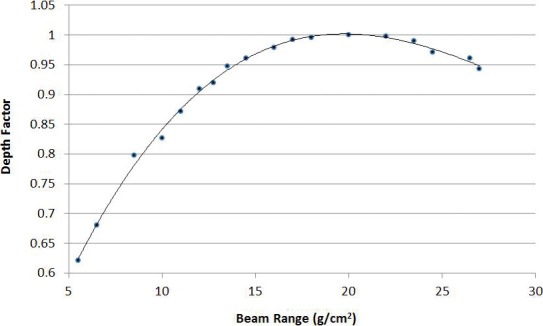
Depth factors as a function of water equivalent beam range.

Relationships between the composite beam range (ie, depth of the target) and MU output were also determined. Depth here is taken to be the average water equivalent depth of the distal surface of the target volume, and the depth factor is defined as the ratio of MU output (cGy/MU) at a given depth relative to the reference depth of 20 cm. This definition of beam range is used because delivered MUs for SFUD-optimized beams are strongly weighted toward the distal end of the target, and it therefore serves as an adequate surrogate for the range of the composite beam. These data are shown in **Figure 3** and were compiled by translating a cubic 125 cm^3^ volume through a water phantom, performing a new optimization at each point. The data were validated for a range of volumes. For depth factors of range-shifted fields, 4.1 cm is added to the calculated water equivalent depth in the phantom to account for the presence of the range shifter.

### Air Gap Factor

**Figure 4.**
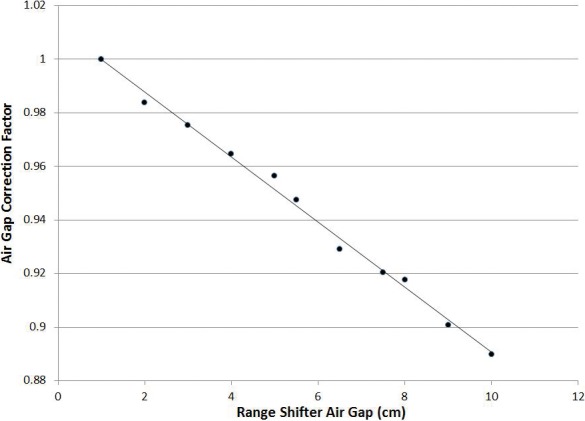
Air gap correction factors as a function of air gap.

For range-shifted fields, output depends on the air gap between the range shifter and the patient. This gap is minimized for dosimetric purposes, but it may be necessary to treat range-shifted fields at larger air gaps owing to physical limitations. The air gap factor corrects the MU output for instances when large air gaps are present for a range-shifted field. It is defined as the ratio of MU output between the given air gap and the reference air gap (1 cm). Data pictured in **Figure 4** were compiled by simulating a cubic 125 cm^3^ volume at 7.5-cm depth (analogous to a typical clinical scenario) in a water phantom, and performing SFUD optimizations for a range of air gaps up to 10 cm. This relationship was spot checked for various other volumes and beam energies, and deemed acceptable.

## Results

**Figure 5.**
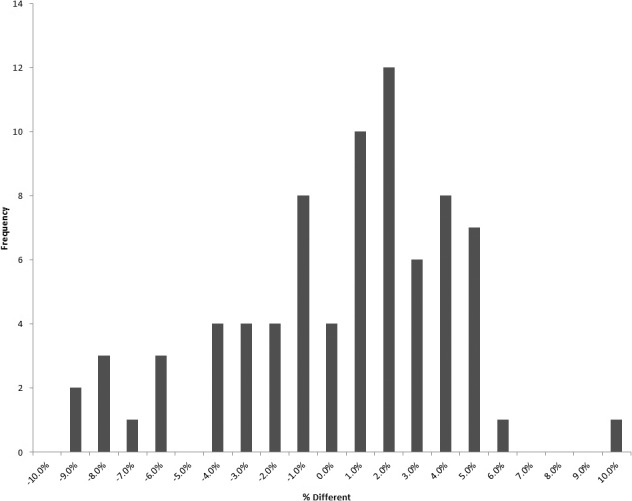
Histogram depicting the percentage difference for all beams.

Our model was applied to various treatment sites including pelvis, brain, lung, and head and neck. Monitor units prescribed by the TPS were compared to those computed by using the proposed method for 78 clinical treatment beams. Results are compiled by site in **Table 1**, as well as in a histogram for all beams in **Figure 5**.

**Table 1.** Results of analysis of difference between TPS-prescribed MU and calculated MU.

**Treatment region**	**Beams**	**Mean difference (%)**	**Standard deviation (%)**
Pelvis	38	0.9	2.1
Brain	13	0.0	4.5
Lung	15	−0.7	4.1
Head and neck	12	−3.1	5.1
Total	78	−0.2	3.8

**Abbreviations:** TPS, treatment planning system; MU, monitor unit.

## Discussion

These results show reasonable agreement between the proposed model and TPS-prescribed MUs for the investigated treatment sites. Good overall mean accuracy of −0.2% is observed with a standard deviation of 3.8%.

It should be noted that the accuracy of the calculation is dependent upon the use of an SFUD optimization technique. Results will be poor if multifield optimization is used, or if organ at risk sparing is prioritized relative to uniform target dose owing to the potentially heterogeneous dose distributions that these scenarios may produce. Moreover, this calculation is dependent upon the overall complexity and achievability of the optimization problem, and is only a practical estimate of the final resulting MUs.

This idea is unique in that it is capable of calculating MUs based only upon the target and beam orientation with respect to the patient, and without detailed spot information. Therefore, this method can be used before or after optimization is performed, as a prediction or verification tool. Furthermore, the overall complexity of this technique is low, reducing the effort required to implement it, and adding a minimal time burden to the clinic workflow.
